# Differentially expressed genes between systemic sclerosis and rheumatoid arthritis

**DOI:** 10.1186/s41065-019-0091-y

**Published:** 2019-06-04

**Authors:** Zhenyu Sun, Wenjuan Wang, Degang Yu, Yuanqing Mao

**Affiliations:** 1 Shanghai Key Laboratory of Orthopaedic Implants, Department of Orthopaedic Surgery, Shanghai Ninth People’s Hospital, Shanghai Jiao Tong University School of Medicine, Shanghai, China; 2 Shanghai Key Laboratory of Orthopaedic Implants, Department of Orthopaedic Surgery, Shanghai Ninth People’s Hospital, Shanghai Jiao Tong University School of Medicine, Shanghai, China

**Keywords:** Gene expression data, Systemic sclerosis, Rheumatoid arthritis, Microarray, Differentially expressed genes, Key genes

## Abstract

**Background:**

Evidence is accumulating to characterise the key differences between systemic sclerosis (SSc) and rheumatoid arthritis (RA), which are similar but distinct systemic autoimmune diseases. However, the differences at the genetic level are not yet clear. Therefore, the aim of the present study was to identify key differential genes between patients with SSc and RA.

**Methods:**

The Gene Expression Omnibus database was used to identify differentially expressed genes (DEGs) between SSc and RA biopsies. The DEGs were then functionally annotated using Gene Ontology (GO) terms and the Kyoto Encyclopedia of Genes and Genomes (KEGG) pathways with the Database for Annotation, Visualization and Integrated Discovery (DAVID) tools. A protein–protein interaction (PPI) network was constructed with Cytoscape software. The Molecular Complex Detection (MCODE) plugin was also used to evaluate the biological importance of the constructed gene modules.

**Results:**

A total of 13,556 DEGs were identified between the five SSc patients and seven RA patients, including 13,465 up-regulated genes and 91 down-regulated genes. Interestingly, the most significantly enriched GO terms of up- and down-regulated genes were related to extracellular involvement and immune activity, respectively, and the top six highly enriched KEGG pathways were related to the same processes. In the PPI network, the top 10 hub nodes and top four modules harboured the most relevant genes contributing to the differences between SSc and RA, including key genes such as *IL6, EGF, JUN, FGF2, BMP2, FOS, BMP4, LRRK2, CTNNB1, EP300, CD79,* and *CXCL13*.

**Conclusions:**

These genes such as *IL6, EGF, JUN, FGF2, BMP2, FOS, BMP4, LRRK2, CTNNB1, EP300, CD79,* and *CXCL13* can serve as new targets for focused research on the distinct molecular pathogenesis of SSc and RA. Furthermore, these genes could serve as potential biomarkers for differential diagnoses or therapeutic targets for treatment.

**Electronic supplementary material:**

The online version of this article (10.1186/s41065-019-0091-y) contains supplementary material, which is available to authorized users.

## Introduction

Systemic sclerosis (SSc) is an autoimmune disease [1] that is often characterised by joint involvement, especially arthritis [2]. However, the degree of synovial inflammation in SSc has not yet been characterised thoroughly [3]. Rheumatoid arthritis (RA) is another autoimmune disease associated with articular damage and consequent disability, which may lead to several complications [4]. The pathogenesis of RA involves excessive reaction of immune components leading to severe inflammation of the joints. Along with recent progress in the diagnosis of SSc and RA based on updated signs and symptoms, several potential diagnostic and therapeutic targets have been uncovered.

In general, SSc and RA are diagnosed by auxiliary approaches such as clinical manifestations, biochemical indicators, and X-ray findings [5, 6]. Since they are both autoimmune diseases with similar clinical signs and symptoms, especially joint involvement, it is not easy to distinguish between them in some cases with uncharacteristic signs and symptoms. To improve the differential diagnosis and therapy of SSc and RA, it is necessary to identify genetic markers that are sufficiently sensitive and highly specific for the two diseases in order to initiate the correct course of treatment.

Gene expression profiling with microarrays is regarded as a standard method for identifying differentially expressed genes (DEGs) and potential biological pathways associated with SSc [7] and RA [8]. To the best of our knowledge, no specific genomic expression analyses have been conducted to distinguish SSc and RA to date. Therefore, in the present study, we investigated the genomic expression profiles to identify DEGs between SSc and RA using a part of the GSE93698 microarray database, including transcriptome data of five SSc tenosynovial biopsy samples and seven RA synovial biopsy samples. Moreover, Gene Ontology (GO) enrichment analyses and Kyoto Encyclopedia of Genes and Genomes (KEGG) pathway analyses were used to perform functional enrichment analysis and identify important biological pathways related to the identified DEGs. In addition, the Retrieval of Interacting Genes (STRING) database was used to construct a protein–protein interaction (PPI) network. Finally, the hub genes of the network were analysed using Cytoscape software, which were used to establish the most significant modules that differentiate SSc and RA.

## Material and methods

### Data source

Gene expression data of seven SSc tenosynovial biopsy samples and five RA synovial biopsy samples were obtained from the Gene Expression Omnibus (GEO) (http://www.ncbi.nlm.nih.gov/geo/) database. The GSE93698 data were derived from the GPL570 microarray platform [HG-U133_Plus_2] Affymetrix Human Genome U133A 2.0 Array.

### Identification of DEGs

Differentially expressed genes (DEGs), including up- and down-regulated genes, were identified between SSc and RA through the R package limma [9, 10] based on the criteria of a statistically significant difference in expression levels (*p* < 0.05) through a t-test [11] and a fold change (FC) > 2. Subsequently, DEGs were ultimately selected according to a false discovery rate < 0.05 and |logFC| > 1.

### Functional enrichment analysis

Gene functional enrichment analyses included classifying gene functions and identifying gene conversions, which were performed through determining enriched Gene ontology (GO) terms [12] and the Kyoto Encyclopedia of Genes and Genomes (KEGG) [13] pathways with the online tool the Database for Annotation, Visualization and Integrated Discovery (DAVID) [14, 15]. Significantly enriched terms/pathways were those with a *P*-value < 0.05 and gene number ≥ 2. In addition, GOplot was used for visualisation of detailed information of the molecules in functional enrichments [16].

### Construction of the PPI network and module analysis

A PPI network was established using the Search Tool for the Retrieval of Interacting Genes (STRING) database [17] based on the significantly up- and down-regulated DEGs to identify the most crucial genes and modules differentiating SSc and RA. A combined score > 0.4 was selected as the cut-off value to construct the PPI network, which was visualised using Cytoscape software. The Molecular Complex Detection (MCODE) plugin was also used to evaluate the biological importance of the constructed gene modules [18]. The top 10 essential nodes ranked by degree were selected, and modules were selected with an MCODE score > 6 and number of nodes > 6.

## Results

### DEGs identification by microarray expression profiling

**Fig. 1:**
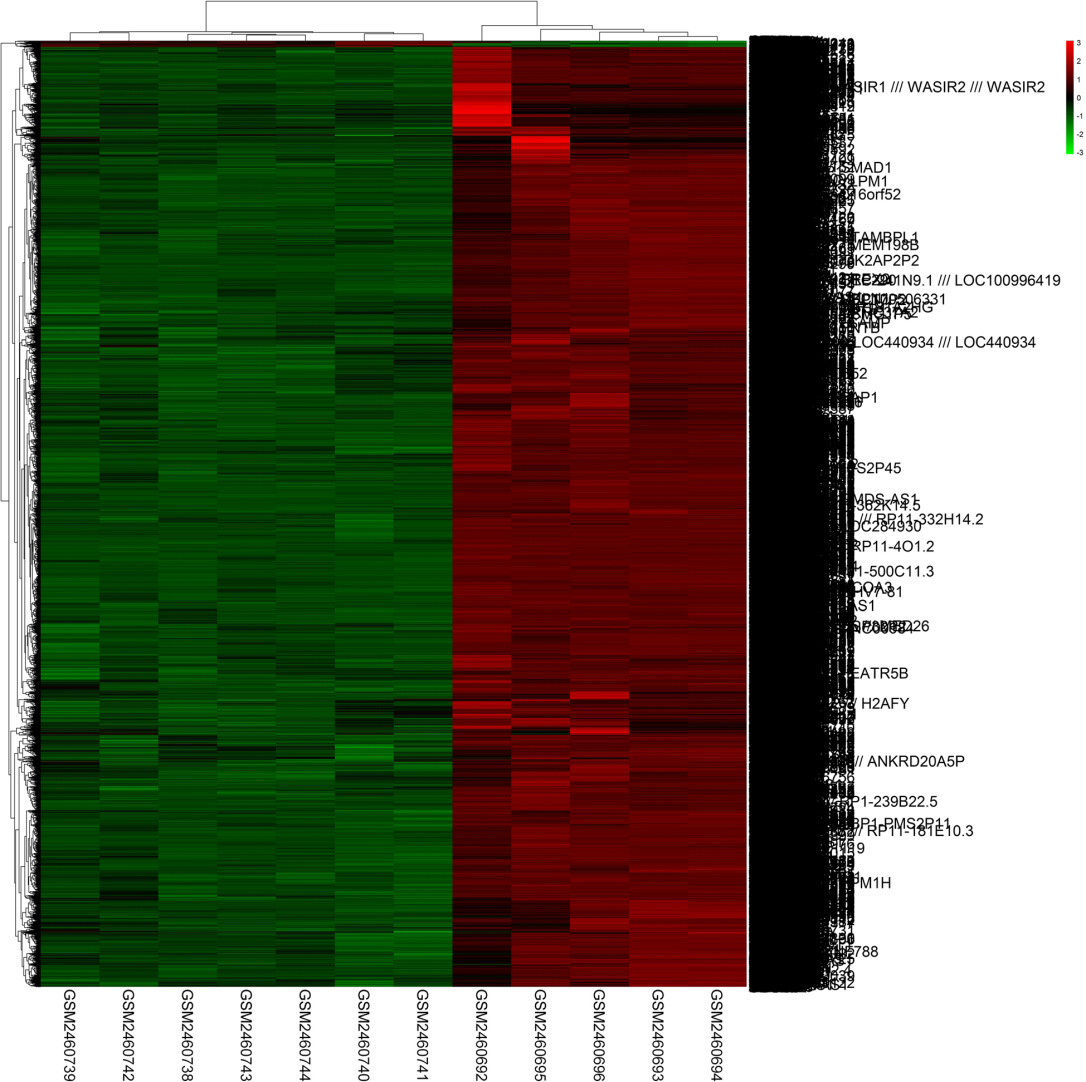
Heatmap of the 13,556 differentially expressed genes (DEGs) between systemic sclerosis and rheumatoid arthritis

Using the GEO GSE93698 dataset of microarray data, we identified a total of 13,556 DEGs (*p* < 0.05 and |logFC| > 1) between SSc and RA samples, including 13,465 up-regulated genes and 91 down-regulated genes. Thus, the great majority of genes that are specifically involved in the SSc pathological process were up-regulated compared to those in RA. The heatmap of the DEGs identified is shown in Fig. 1.

### GO functional enrichment

**Fig. 2:**
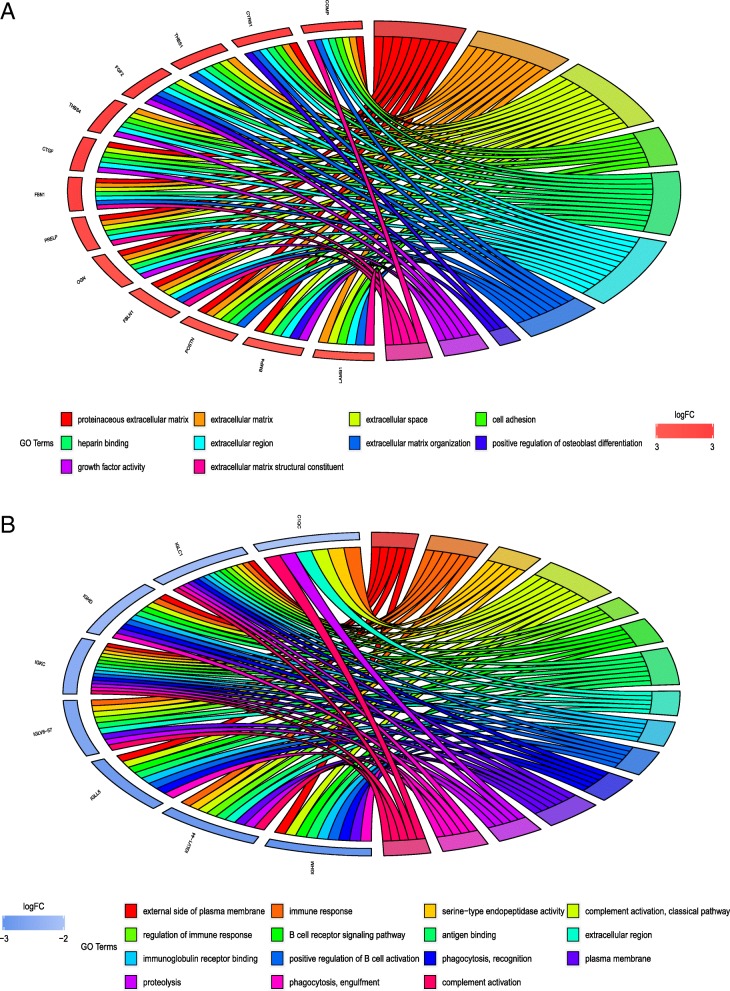
Gene Ontology enrichment analyses. **a** Gene Ontology enrichment of up-regulated genes. **b** Gene Ontology enrichment of down-regulated genes. The criteria for enrichment were: *P*-value < 0.05, FDR < 0.05, and fold enrichment > 1. Each gene was assigned to at least six terms

To investigate the functions of the large range of gene signatures obtained, we performed GO enrichment analysis from the GO database [19] including terms of the biological process, molecular function, and cellular component categories for the top 1000 up-regulated genes and 91 down-regulated genes (Fig. 2).

Ten terms were enriched for the up-regulated genes, which were predominantly related to extracellular activities in the biological process, molecular function, and cellular component categories. In the cellular component cluster, representative terms were related to proteinaceous extracellular matrix (ECM), ECM, extracellular space, and extracellular region. In addition, the biological process cluster included terms of cell adhesion, ECM organization, and positive regulation of osteoblast differentiation. The significantly enriched terms for the molecular function cluster were heparin binding, growth factor activity, and ECM structural constituent. The up-regulated genes *COMP, CYR61, THBS1, FGF2, THBS4, CTGF, FBN1, PRELP, OGN, FBLN1, POSTN, BMP4*, and *LAMB1* appeared frequently in these terms (Fig. 2). However, the 15 enrichment terms related to the down-regulated genes were mainly related to immune activities, which could have important clinical implications. These representative down-regulated genes enriched in these terms were *C1QC, IGLC1, IGHD, IGKC, IGLV6–57, IGLL5, IGLV1–44*, and *IGHM*.

### KEGG pathways analysis

KEGG pathway analysis identified the top six important KEGG pathways of the up- and down-regulated DEGs (Table 1), including ECM–receptor interaction, Wnt signalling pathway, transforming growth factor-beta (TGFβ) signalling pathway, primary immunodeficiency, hematopoietic cell lineage, and cytokine–cytokine receptor interaction.

**Table 1:** Top 3 the most significantly enriched KEGG pathways of up and down regulated DEGs respectively

Regulate	Term	Count	*P*-value	Genes	Fold Enrichment
up	hsa04512:ECM-receptor interaction	16	4.35E-06	COL4A4, ITGA1, ITGA2, ITGA10, CHAD, LAMA2, CD36, COMP, RELN, TNN, THBS1, LAMB1, COL24A1, THBS2, COL11A1, THBS4	4.194075
up	hsa04310:Wnt signaling pathway	19	2.80E-05	VANGL2, MMP7, PPP3R1, FZD7, FZD6, CTNNB1, WNT2, GPC4, PLCB4, EP300, DKK1, PRICKLE1, SFRP2, JUN, SFRP4, PRICKLE2, WNT9A, SOX17, BAMBI	3.139857
up	hsa04350:TGF-beta signaling pathway	14	6.24E-05	BMP4, BMP2, FST, BMPR2, SMAD1, ACVR1C, INHBA, EP300, ID1, BAMBI, THBS1, BMPR1B, BMP5, ACVR1	3.80088
down	hsa05340:Primary immunodeficiency	6	3.89E-07	CD19, CD3D, CD8A, IL2RG, CD79A, IL7R	36.95187
down	hsa04640:Hematopoietic cell lineage	7	2.03E-06	CD19, CD3D, CD8A, MS4A1, CD2, IL7R, CSF1R	17.24421
down	hsa04060:Cytokine-cytokine receptor interaction	6	0.003777	CXCL13, IL21R, TNFRSF17, IL2RG, IL7R, CSF1R	5.462451

*KEGG* Kyoto encyclopedia of genes and genomes, *DEGs* differentially expressed genes

### Construction of the PPI network and module analysis

**Fig. 3:**
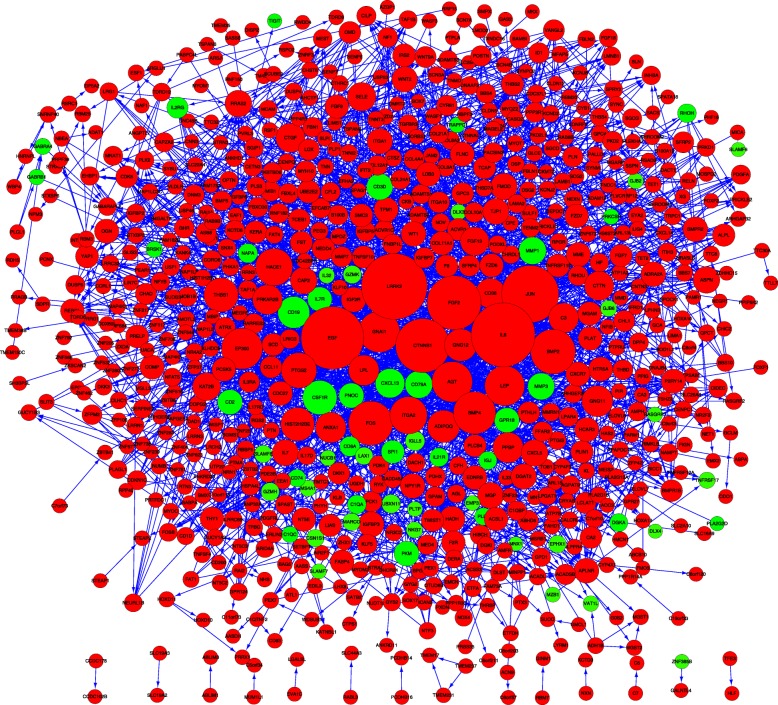
Protein–protein interaction network constituted by differentially expressed genes identified in this study. Red nodes represent up-regulated genes, and green nodes represent down-regulated genes

The top 1000 up-regulated genes and 91 down-regulated genes were mapped by the STRING database to establish a PPI network (Fig. 3). Protein pairs with a combined score of > 0.4 were selected. According to the information from STRING, the top 10 hub nodes with high degrees were identified using the Cytoscape tool, including *LRRK2, IL6, EGF, JUN, CTNNB1, FGF2, BMP2, FOS, BMP4*, and *EP300* (Additional file 1: Table S1). The largest node degrees were detected for *CXCL5, CXCL13, GPR18, NPY1R, ADRA2A, CXCR7, AGT, GNAI1, HTR5A, HCAR3, GNG11, P2RY14, ANXA1, PPBP, C3, PNOC, GNG12*, and *APLNR* (Additional file 2: Table S2), suggesting that these genes may play an important role in the pathological process.

**Fig. 4:**
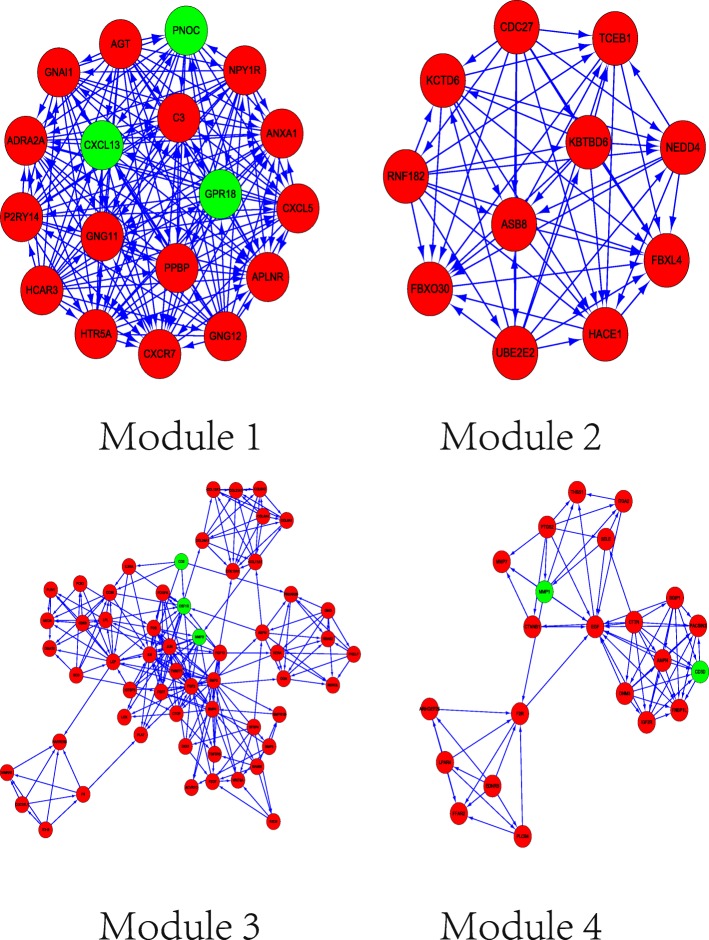
Four significant modules identified from the protein–protein interaction network using the molecular complex detection method with a score > 6.0: Module 1 (MCODE score = 18), Module 2 (MCODE score = 11), Module 3 (MCODE score = 8.035), and Module 4 (MCODE score = 7.048)

The top four modules of the PPI network are presented in Fig. 4. Enrichment analyses of these modules (Table 2) showed that genes in module 1 (including *PPBP, CXCL13, GNG12, CXCL5, HTR5A, GNAI1, P2RY14, HTR5A,* and *ADRA2A*) were associated with chemokine signalling pathway, serotonergic synapse, and neuroactive ligand-receptor interaction. Module 2 included *NEDD4, CDC27, UBE2E2*, and *TCEB1* associated with ubiquitin-mediated proteolysis and renal cell carcinoma pathways. In addition, pathways in cancer, signalling pathways regulating stem cells, and the Hippo signalling pathway were central components of module 3, which was enriched in the key genes *JUN, WNT9A, FGF2, BMP4, BMP2, IL6, COL4A4,* and *FGF13*, among others. Finally, module 4 included the genes *PLCB4, MMP1, CTNNB1, EGF, F2R*, and *LPAR4*, associated with pathways in cancer, Rap1 signalling pathway, and phospholipase D signalling pathways.

**Table 2:** KEGG pathway enriched by differentially expressed genes in different modules

Term	Description	*P*-value	Genes
Module1			
hsa04062	Chemokine signaling pathway	1.04E-08	PPBP;CXCL13;GNG12;GNG11;CXCL5;GNAI1
hsa04726	Serotonergic synapse	2.68E-06	HTR5A;GNG12;GNG11;GNAI1
hsa04080	Neuroactive ligand-receptor interaction	3.63E-06	P2RY14;NPY1R;APLNR;HTR5A;ADRA2A
Module2			
hsa04120	Ubiquitin mediated proteolysis	6.7E-07	NEDD4;CDC27;UBE2E2;TCEB1
hsa05211	Renal cell carcinoma	0.035716	TCEB1
Module3			
hsa05200	Pathways in cancer	5.39E-12	CSF1R;JUN;FZD7;FZD6;PDGFA;WNT9A;FOS;FGF2;BMP4;FGF7;BMP2;IL6;COL4A4;FGF13
hsa04550	Signaling pathways regulating pluripotency of stem cells	7.52E-09	BMP4;BMP2;ACVR1C;FZD7;FZD6;WNT9A;BMPR1B;FGF2
hsa04390	Hippo signaling pathway	1.35E-08	BMP4;BMP2;FZD7;FZD6;WNT9A;BMPR1B;CTGF;BMP5
Module 4			
hsa05200	Pathways in cancer	1.73E-10	PLCB4;EDNRB;EGF;MMP1;ITGA2;F2R;CTNNB1;LPAR4;PTGS2
hsa04015	Rap1 signaling pathway	8.32E-08	PLCB4;EGF;F2R;CTNNB1;LPAR4;THBS1
hsa04072	Phospholipase D signaling pathway	4.31E-07	DNM3;PLCB4;EGF;F2R;LPAR4

Top 3 terms were selected according to *P*-value when more than 3 terms enriched terms were identified in each category

## Discussion

Despite progress in the differential diagnoses between SSc and RA, a more effective and sensitive method for helping to distinguish between these two diseases is needed. The present findings highlight some distinct pathological molecular mechanisms of these two diseases, which may provide further information as therapeutic targets. Of the 13,556 DEGs including 13,465 up-regulated genes and 91 down-regulated genes identified between SSc and RA samples. Interestingly, the number of up-regulated genes were far more than the number of down-regulated genes, which was related to huge different characteristics between these two different diseases. The majority were up-regulated in SSc; these genes were mainly related to extracellular activities, which should be taken into account in further studies on SSc pathology. SSc is an autoimmune rheumatic disease with multisystem fibrosis manifestations. In normal physiological conditions, fibroblasts can be protected by the ECM, whereas the damaged fibroblasts in SSc attached to the ECM are destroyed [20]. Abnormal ECM remodelling mechanisms are linked to the fibrosis that occurs in connective tissue diseases. Excessive ECM, including collagens, hyaluronic acid, fibronectin, and proteoglycans, promotes scarring and sustained fibrosis, leading to excessive scar tissue [21]. Some studies suggested that overexpression of ECM genes may have a central effect in fibrotic cells [22–24]. Pathologically activated fibrosis arises from high accumulation of ECM components, highlighting a novel therapeutic approach in SSc [25]. However, we further showed that many of the down-regulated genes in SSc were related to immune activities, which warrants further investigation to identify new therapeutic targets. Indeed, the pathological process of RA is well known to be related to an excessive or dysregulated immune response, including an abnormal autoimmune response and genetic susceptibility. Immune cells such as dendritic cells, T cells, B cells, and natural killer cells are all related to the development of RA [26]; thus, genes regulating immune activities have been highlighted as potential therapeutic targets for RA [27]. For example, anti-CD79A antibody therapy was shown to enhance immune system recovery against autoimmunity [28]. Thus, our results further indicate that fibrosis and other clinical manifestations may be related to the immune response.

KEGG pathway enrichment analyses of the identified DEGs demonstrated six key significantly enriched pathways, including ECM–receptor interaction and primary immunodeficiency, which is in line with the known pathological mechanisms. However, the other pathways identified, including Wnt signalling pathway, TGFβ signalling pathway, hematopoietic cell lineage, and cytokine–cytokine receptor interaction, deserve new attention. The selective stabilization of β-catenin leads to excessive ECM production [29], and Wnt may induce the fibroblast activation and abundant collagen production related to SSc [30]. Indeed, the Wnt pathway has been proposed to be a core factor involved in the progression of SSc [30]. Moreover, the Wnt cascade tightly interacts with TGFβ signalling, which may be involved in ECM activities [31]. Cells of the hematopoietic lineage such as CD79a-positive B cells are helpful for diagnosing RA [32], and this pathway has deep associations with many immune hematopoietic cells. Similarly, the cytokine–cytokine receptor interaction is another important component of the RA pathological mechanism [33]. Therefore, monitoring these signalling pathways may aid in the prediction of the progression of these two diseases.

The PPI network constructed with the DEGs resulted in 10 hub genes that can be used to differentiate between SSc and RA: *LRRK2, IL6, EGF, JUN, CTNNB1, FGF2, BMP2, FOS, BMP4,* and *EP300*. These genes have also been previously highlighted to play a role in the pathogenesis of SSc and RA. IL-6, which is linked to the ECM, can regulate collagen synthesis by fibroblasts in SSc [34, 35], and EGF was shown to up-regulate TGFRII expression in SSc fibroblasts [36]; thus, EGFR signalling was suggested as a therapeutic target in fibrotic diseases [37]. FGF-2 plays a role in the pathogenic process of pulmonary arterial hypertension, which modifies pulmonary vascular remodelling leading to the vascular manifestations in SSc [38]. FGF, which regulates the synthesis of collagen and ECM components, is up-regulated by TGFβ in SSc, resulting in an increase in BMP signalling [39]. The AP-1 family members c-Jun, c-Fos, and JunD are also known to play an important role in SSc [40, 41]. JunD is a downstream mediator of TGFβ signalling [40], and Jun N-terminal kinases are regarded as intracellular mediators that may be affected by TGFβ [41]. Thus, almost all of these genes are associated with TGFβ, which is known to play a key role in fibrotic diseases. LRRK2 [42] and CTNNB1 [43] were also reported to interact with the Wnt pathway in fibromatosis. However, EP300 is mainly known to influence specific T cell states [44]. Although *LRRK2, CTNNB1*, and *EP300* have not been previously clearly associated with SSc, our results suggest that they may be potential biomarkers of this disease, and thus worthy of further investigation.

Module analyses of the PPI network further revealed the differential development of SSc and RA. *CXCL13* in module 1, related to the chemokine signalling pathway, has been linked to joint inflammation and the development of autoimmune disorders, including RA [45]. CXCL13 is a proinflammatory cytokine that can serve as a biomarker in early RA and reflects the severity of synovitis [46]. Thus, CXCL13 appears to represent a new direction for RA treatment. *FGF2* and *IL6* in module 3 are regarded as important factors for SSc development based on the background summarised above, as is *EGF*, part of module 4, which mediates the up-regulation of TGFβ receptor in SSc. However, many genes identified in this study have not been previously associated with SSc or RA, such as *NEDD4, CDC27,* and *UBE2E2* in module 2. Thus, attention should be focused on these genes in future work as potential biomarkers and therapeutic targets.

## Conclusions

This study identified a series of core genes and pathways that differentiate SSc and RA. Compared to RA, the majority of the up-regulated genes in SSc were related to extracellular activities, whereas the down-regulated genes were mainly related to immune activities. These two distinct processes can provide a new direction for methods of clinically distinguishing between these two diseases by focusing on the involvement of extracellular and immune activities. On the one hand, *IL6, EGF, JUN, FGF2, BMP2, FOS,* and *BMP4* should be further studied as they may play an essential and specific role in the SSc pathogenesis. On the other hand, *CD79* and *CXCL13* might be representative genes for RA. These genes might be used as biomarkers to improve the differential diagnosis and treatment of SSc and RA. However, many of the other genes identified in this study have not been previously reported to be associated with these diseases, such as *LRRK2, CTNNB1*, and *EP300*, and should not be ignored. Thus, further studies and clinical trials are needed to verify our findings and establish reliable diagnostic or therapeutic targets.

## Additional files

Additional file 1: **Table S1.** Degree of top 10 genes. (DOCX 14 kb)

Additional file 2: **Table S2.** The modules of the PPI network. Four modules from the protein-protein interaction network satisfied the criteria of MCODE scores >6 and number of nodes >6. (DOCX 14 kb)

## Abbreviation

DAVID: the Database for Annotation, Visualization and Integrated Discovery
DEG: Differentially expressed gene
ECM: Extracellular matrix
GO: Gene Ontology
KEGG: Kyoto Encyclopedia of Genes and Genomes
MCODE: the Molecular Complex Detection
PPI: Protein–protein interaction
RA: Rheumatoid arthritis
SSc: System sclerosis
STRING: the Search Tool for the Retrieval of Interacting Genes
TGFβ: Transforming growth factor-beta
