# Supplementary material for: Differentially expressed genes between systemic sclerosis and rheumatoid arthritis
Source: Hereditas. 2019 Jun 4;156:17. doi: 10.1186/s41065-019-0091-y (PMC6549285; doi:10.1186/s41065-019-0091-y)
Supplement: Supplementary file 1 — Table S1. Degree of top 10 genes. (DOCX 14 kb) [file 41065_2019_91_MOESM1_ESM.docx]

Table S1. Degree of top 10 genes.

| Gene ID | Gene name | Degree | Expression |
| --- | --- | --- | --- |
| LRRK2 | leucine rich repeat kinase 2 | 84 | up |
| IL6 | interleukin 6 | 81 | up |
| EGF | epidermal growth factor | 79 | up |
| JUN | Jun proto-oncogene | 70 | up |
| CTNNB1 | catenin beta 1 | 65 | up |
| FGF2 | fibroblast growth factor 2 | 65 | up |
| BMP2 | bone morphogenetic protein 2 | 50 | up |
| FOS | Fos proto-oncogene | 49 | up |
| BMP4 | bone morphogenetic protein 4 | 46 | up |
| EP300 | E1A binding protein p300 | 44 | up |
