# Supplementary material for: Differentially expressed genes between systemic sclerosis and rheumatoid arthritis
Source: Hereditas. 2019 Jun 4;156:17. doi: 10.1186/s41065-019-0091-y (PMC6549285; doi:10.1186/s41065-019-0091-y)
Supplement: Supplementary file 2 — Table S2. The modules of the PPI network. Four modules from the protein-protein interaction network satisfied the criteria of MCODE scores >6 and number of nodes >6. (DOCX 14 kb) [file 41065_2019_91_MOESM2_ESM.docx]

Table S2. The modules of the PPI network. Four modules from the protein-protein interaction network satisfied the criteria of MCODE scores >6 and number of nodes >6.

| Cluster | Score (Density*#Nodes) | Nodes | Edges | Node IDs |
| --- | --- | --- | --- | --- |
| 1 | 18 | 18 | 153 | CXCL13, GPR18, AGT, NPY1R, ADRA2A, CXCR7, ANXA1, GNAI1, GNG12, HTR5A, HCAR3, GNG11, CXCL5, P2RY14, C3, PPBP, PNOC, APLNR |
| 2 | 11 | 11 | 55 | TCEB1, ASB8, KCTD6, FBXL4, HACE1, UBE2E2, CDC27, NEDD4, FBXO30, RNF182, KBTBD6 |
| 3 | 8.035 | 58 | 229 | OMD, CD2, WNT9A, KERA, FZD7, CSF1R, RRAS2, MMRN1, PRKAR2B, DKK1, IL6, BMP2, F8, SFRP4, COL8A2, BMP4, CDC37L1, MED4, COL12A1, CDK8, BMP5, COL10A1, COL11A1, ITIH3, RERGL, FGF13, PDGFA, RARRES2, PCK1, BMPR1B, TWIST1, ASPN, LOX, FGF2, PLIN1, COL21A1, FGF7, LPL, CTGF, COL4A4, SCD, COL24A1, TGFBR3, PRELP, CD36, MMP3, PLAT, ACVR1C, FZD6, LEP, DGAT2, IL3RA, BAMBI, FOS, JUN, COL8A1, IGFBP3, OGN |
| 4 | 7.048 | 22 | 74 | SELE, CTTN, PTGS2, IGF2R, ITGA2, FFAR2, PACSIN3, LPAR4, AMPH, EGF, FNBP1L, DNM3, CTNNB1, SGIP1, PLCB4, ARHGEF25, THBS1, EDNRB, F2R, MMP1, CD3D, MMP7 |
